# Development and validation of a French job-exposure matrix for healthcare workers: JEM Soignances

**DOI:** 10.5271/sjweh.4194

**Published:** 2024-12-01

**Authors:** Allison Singier, Marc Fadel, Fabien Gilbert, Soignances group, Ester-MESuRS collaboration on occupational risks, Laura Temime, Marie Zins, Alexis Descatha

**Affiliations:** 1 Univ Angers, Univ Rennes, Inserm, EHESP, Irset (Institut de recherche en santé, environnement et travail) - UMR_S 1085, SFR ICAT, Angers, France.; 2 Univ Angers, CHU Angers, Univ Rennes, Inserm, EHESP, Irset (Institut de recherche en santé, environnement et travail) - UMR_S 1085, SFR ICAT, Angers, France.; 3 Inserm, Université Paris Cité, Université Paris Saclay, Université de Versailles-Saint-Quentin-en-Yvelines (UVersusQ), UMS 11 “Population-based Epidemiological Cohorts Unit”, UMS 011, Villejuif, France.; 4 Modélisation, épidémiologie et surveillance des risques sanitaires (MESuRS), Conservatoire national des arts et métiers, Paris, France.; 5 CHU Angers, Poisoning Control Center, Federation of Prevention, Angers, France.; 6 Department of Occupational Medicine, Epidemiology and Prevention, Hofstra-Northwell Health, New York, USA.; * Soignances group: Yves Roquelaure, Laurent Poiroux (Inserm Irset/Univ Angers CHU Angers, Angers), Annette Leclerc, Marcel Goldberg (Inserm UMS011/Université de Paris, Villejuif), William Dab, Mohamed Ben Halima, Kevin Jean (CNAM, Paris), Cédric Lemogne, Guillaume Airagnes (AP-HP, Université Paris Cité, Paris), Pascal Guénel, Marina Kvaskoff (Inserm CESP, Villejuif), Sandrine Caroly (Université Grenoble Alpes, Grenoble).; ** Ester-MESuRS collaboration on occupational risks: Alexis Descatha, Marc Fadel, Yves Roquelaure, (Inserm Irset/Univ Angers CHU Angers, Angers) and Laura Temime, William Dab, Mohamed Ben Halima, Kevin Jean (CNAM, Paris).

**Keywords:** caregiver, CONSTANCES, exposome, exposure assessment, HCW, health professional, occupational

## Abstract

**Objectives:**

This study aimed to develop and evaluate a job-exposure matrix (JEM) specific to healthcare workers, JEM Soignances, based on self-reported data.

**Methods:**

The JEM was constructed using data from healthcare workers within the CONSTANCES cohort (N=12 489). Job titles and sectors of activity (eg, hospital activities) defined occupational groups. We assessed 24 exposures covering organizational, psychosocial, physical, chemical and biological factors. Several methods (group-based frequency, CART, random forest, extreme gradient boosting machine) were applied using a 70% training sample. Performance was evaluated on the remaining 30% using area under the ROC curve (AUC) and Cohen’s Kappa (κ). Two alternative JEM were proposed using only job titles or adding healthcare establishment size and type (public/private) to define occupational groups.

**Results:**

All methods offered similar discriminatory power (AUC). We selected the group-based frequency method as it was the most understandable and easiest to implement. Of the 24 included exposures, 15 demonstrated satisfactory performance, with nine showing good discriminatory power and fair-to-moderate agreement, such as *physical effort at work* (AUC=0.861,κ=0.556), *ionizing radiation exposure* (AUC=0.865,κ=0.457), *carrying heavy loads* (AUC=0.840,κ=0.402), s*hift work* (AUC=0.807,κ=0.383), and *formaldehyde exposure* (AUC=0.847,κ=0.289). The remaining nine exposures mainly showed poor-to-moderate discriminatory power and poor agreement. Compared to JEM Soignances, the job title-only JEM performed poorly, while the one incorporating healthcare establishment size and type showed similar results.

**Conclusions:**

JEM Soignances provides good internal performance and validity. Future research will assess its external validity by comparing it with existing JEM and examining its predictive validity regarding known associations between exposures and health outcomes (eg, long working hours and strokes).

In recent years, the health and well-being of healthcare workers has received increased attention, a trend amplified by the COVID-19 pandemic. Healthcare workers play a critical role in delivering high-quality healthcare services and maintaining the sustainability of the whole healthcare system. Consequently, governments worldwide have instituted policies and initiatives to prioritize the enhancement of healthcare workers’ health and safety (1). Notably, England’s ‘Our NHS People’ campaign has been launched to support and empower healthcare professionals within the National Health Service (NHS) along with the establishment of specialized services aimed at addressing their mental health needs (2, 3). Similarly, Canada has made notable financial investments in addressing mental health challenges among healthcare workers (4, 5). Furthermore, countries like Finland, Sweden, and Spain have implemented various policies and decrees aimed at preventing and controlling occupational risks and improving working conditions for healthcare workers (6, 7).

The demanding nature of healthcare workers’ jobs exposes them to various occupational hazards, including long working hours and night shifts, carrying heavy loads and ergonomic risks, and exposure to infectious diseases, all of which pose important threats to their physical and mental well-being. Additionally, organizational factors, such as job strain, lack of social support, and inadequate resources, contribute to the overall burden of occupational stress experienced by these professionals. However, studies on healthcare workers often focus on individual exposures, lacking a comprehensive tool to assess multiple occupational factors concurrently, or exposures occurring in the past. In this context, job-exposure matrices (JEM) can be seen as valuable tools to assess occupational exposures based on job titles, tasks, and environmental factors. Widely used in occupational epidemiology research to estimate workers’ exposures to chemical or physical risk factors, JEM are constructed from diverse data sources including exposure measurements, observations of workers, expert knowledge, and self-reported exposures (8). JEM focusing on specific sectors have been developed (9–11), but none allow for the comparison of exposures across different work situations within the healthcare sector. In this context, this study aimed to develop and evaluate a specific JEM for healthcare workers occupational risk factors, based on self-reported data.

## Methods

### Data source and population

For this study, data from the French CONSTANCES population-based cohort were used (doi.org/10.13143/inserm_constances). CONSTANCES is designed to study occupational and social determinants of health and includes around 220 000 volunteers recruited between 2012 and 2021, aged 18–69 years, and affiliated to the French general health plan. Participants were randomly recruited from 21 selected national health insurance medical screening centers across France (more details at: www.constances.fr) (12, 13). Among them, only participants with ≥6 months’ work experience were considered (N=182 007). The Soignances cohort was then constituted of all healthcare workers (including veterinarians) who were currently working and whose job could be coded based on their questionnaire responses (N=12 489) (14).

### Job title and sector of activity

Each job title was assigned a 4-digit code corresponding to the last level of the French classification of occupations (three-level nested occupational classification system; *Professions et categories sociales,* PCS 2003) (15). A crosswalk between PCS-2003 and the International Standard Classification of Occupations (ISCO-2008) is provided in supplementary material (www.sjweh.fi/article/4194), table S1. To account for variations across healthcare workers sectors of activity (eg, hospital activities, nursing care facilities), a code was assigned according to the French equivalent of the European Nomenclature of Economic Activities (NACE) classification (five nested levels classification; *Nomenclature des activités françaises*, NAF-2008) (16). Depending on the information available, a 1-character code corresponding to the least detailed section of the sector of activity up to a 5-digit code corresponding to the most detailed sector of activity was assigned. In this study, 31 PCS codes specific to health professions were identified, with a total of 144 associated NAF codes. These PCS+NAF groupings will be referred to as occupational groups.

### Exposure measures

The CONSTANCES questionnaires assessed a wide range of exposures, 24 of which were considered relevant for healthcare activities in the development of JEM Soignances. These exposures were assessed at the time of inclusion and mainly deal with organizational constraints, biomechanical, physical, chemical, biological, and psychosocial factors. Details about exposure assessment are available in table 1. For 17 of these, the presence or absence of exposure was reported as a binary variable, such as *long working hours*, exposure to *ionizing radiation*, or *infectious risks*. For the remaining 7 exposures, different methods were used to assess exposure: 4 were assessed using a 4-point Likert scale, with response options ranging from ‘never or nearly never’ to ‘always or nearly always’, such as *carrying heavy loads (>25kg)*, or *arms above shoulders* and *kneeling* postures. The level of *physical effort at work* was assessed for the working participants using a scale ranging from 0 (sedentary) to 3 (heavy efforts). Overall intensity of physical effort at work was assessed using the Borg Rating of Perceived Exertion Scale, which ranges from 6 (no effort required) to 20 (exhausting). Finally, *effort-reward imbalance (ERI)* was assessed using a calculated score, based on three effort items and seven reward items from the French version of the short ERI questionnaire, as referred to by Siegrist (17, 18). To harmonize the type of exposure variables, ordinal and continuous exposures were dichotomized. For the 4-point Likert scale variables, ‘often’ and ‘always or nearly always’ were considered to indicate exposure, whereas ‘rarely’ and ‘never or nearly never’ were considered to indicate non-exposure. For *physical effort at work*, only participants reporting heavy efforts were considered as being exposed. For the Borg Rating of Perceived Exertion Scale, a value ≥13 was used to indicate *intense physical effort*. For the *effort-reward imbalance* variable, a calculated score ≥1.5 was used to indicate high effort-reward imbalance.

**Table 1:** Details on the evaluation and use of the 24 self-reported exposures of interest for JEM Soignances’ development. [JEM=job exposure matrix.]

Exposure		Description	Scale (cut-off) ^a^	Simplified JEM		JEM Soignances		Alternative JEM
				N group/N ^b^		N group/N ^b^		N group/N ^b^
**Organizational constraints**								
	Late hours (bedtime after midnight)	Do you have (or have you had) work and travel times requiring you to go to bed after midnight at least 50 days per year?		30/12 372		117/11 335		153/8537
	Early hours (up before 5am)	Do you have (or have you had) work and travel times requiring you to get up before 5am at least 50 days per year?		30/12 369		117/11 334		153/8544
	Sleepless nights	Do you have (or have you had) work and travel times requiring you not to sleep at night at least 50 days per year?		30/12 407		117/11 375		153/8576
	Long working hours (>10h)	Do you have (or have you had) a daily work time (excluding travel) of more than 10 hours at least 50 days per year?		30/12 383		117/11 354		152/8555
	Weekly rest <48h consecutive	Do you regularly have (or have you had) less than 48 consecutive hours of rest per week?		30/12 318		116/11 278		153/8513
	Shift work	Do you have (or have you had) an alternating times shift-based job (teams, brigades, rotations, etc.)?		30/12 312		117/11 286		151/8493
	Saturday work (more than one in two)	Do you work (or have you worked) more than one in two Saturdays during the year?		30/12 403		117/11 371		152/8566
	Sunday work (more than one in two)	Do you work (or have you worked) more than one in two Sundays during the year?		30/12 391		117/11 368		153/8566
**Biomechanical factors**								
	Time-constrained job	Do you have (or have you had) a repetitive and time-constrained job (line production work, moving product or parts, automatic rate machine, rate imposed by strict standards, etc.)?		30/12 382		117/11 350		153/8568
	Repetitive work	During a typical working day: Do you need to repeat the same actions more than 2 to 4 times per minute?	Likert 1–4 (3)	30/12 022		116/10 998		151/8381
	Physically difficult work	During your professional life, have you been (or are you currently) exposed to physically difficult work?		30/12 299		116 /11 268		152/8492
	Physical effort at work	For working participants: In your current job, what level of physical effort is required of you?	0–3 (3)	30/11 970		115 /10 958		149/8242
	Carry heavy loads	During your professional life, have you been required (or are you currently required) to carry heavy loads?		30/12 235		117/11 296		152/8515
	Carry heavy loads (>25kg)	How much time do you spend performing the following tasks or activities? Carrying a load weighing more than 25 kg	Likert 1–4 (3)	30/11 978		117/10 973		148/8277
	Arms above shoulder	During a typical working day, during how much time do you have to adopt the following positions: Work with one or both arms raised (above the shoulders) on a regular or prolonged basis?	Likert 1–4 (3)	30/12 203		117/11 183		150/8430
	Kneel or squat	On a typical working day: Do you need to kneel or crouch?	Likert 1–4 (3)	30/12 285		117/11 258		151/8551
	Intense physical effort (Borg)	How would you rate the intensity of physical effort during a typical working day?	6–20 (13)	30/12 187		116/11 151		151/8476
**Physical factors**								
	Noise pollution	Do you work (or have you worked) in an environment occasionally requiring you to raise your voice to be heard by a person located less than 2 or 3 metres from you?		30/12 411		117/11 380		153/8584
	Noisy tools	Do you work (or have you worked) with or in the vicinity of noisy tools, machines or vehicles?		30/12 385		117/11 353		153/8563
	Ionizing radiation	Are you, or have you been during your professional life, exposed to ionizing radiation (X-rays, gamma rays, etc.)?		30/11 900		109/10 820		146/8153
**Biological and chemical factors**								
	Formaldehyde	During your professional life, have you been (or are you currently) exposed to formaldehyde?		30/12 001		114/10 963		146/8211
	Infectious risks	Does (or did) your work or workplace present an infectious risk (micro-organisms, viruses, parasites, etc.)?		30/12 064		114/11 051		151/8385
	Live or dead animals	Are you, or have you been during your professional life, in contact with live or dead animals?		30/12 244		116/11 217		150/8452
**Psychosocial factors**								
	Effort-reward imbalance	Calculated	0.33–4 (1.5)	30/12 038		115/11 018		148/8288

^a^ Scale of initial data for non-binary variables and cut-off used for dichotomization; Likert 1–4: “Never or nearly never”, “rarely”, “often”, “always or nearly always”; 0–3: Sedentary, light effort (walking and carrying loads <10kg), moderate effort (carrying loads 10–25kg), heavy effort (carrying loads >25kg). ^b^ N group / N: number of occupational groups (4-digits PCS, 4-digits PCS/ 5-digits NAF, and 4-digits PCS/ 5-digits NAF/ healthcare establishment size/ healthcare establishment type for simplified JEM, JEM Soignances and alternative JEM respectively) / number of individuals.

### JEM development and internal validity assessment

*JEM Soignances.* The JEM Soignances (short for *‘soignants dans Constances’*) was developed to assess occupational exposures among healthcare workers in France when only job data are available. To ensure reliable estimates, for each exposure, occupational groups with ≤10 participants were excluded from data analysis. Multiple methods were used to estimate the JEM’s exposure probabilities of healthcare professionals for the 24 pre-identified factors. To compare the performance of these methods under identical conditions and avoid overfitting, the data were randomly divided into two datasets: a learning dataset accounting for 70% of the total dataset, used to calculate probabilities, and a test dataset accounting for the remaining 30%, used to evaluate the performance of the method by comparing results obtained through calculated probabilities and self-reported data.

The first method, which we called “group-based frequency”, is comparable to those of JEM previously developed in Norway, Finland, or France, except that the performances were assessed in a different sample than the one used to develop the JEM (19–21). First, for each exposure of interest, the frequency of participants exposed, which corresponds to the JEM exposure assessment, was calculated for every occupational group using the learning sample. Second, the calculated frequencies were applied to the test sample, dichotomized, and then compared to the individually self-reported exposures. Specifically, if the frequency exceeded a pre-specified threshold, all the participants within this occupational group were considered exposed, otherwise, the group was classified as unexposed. The results were computed using different pre-specified thresholds (ranging from 0 to 1 in increments of 0.05) to calculate the following performance indicators: Cohen’s Kappa (κ), specificity, sensitivity, and F1-Score. Cohen’s Kappa was used to measure the strength of agreement between the group-based frequency method and self-reported exposure and was interpreted as follows: poor (0–0.20), fair (0.21–0.40), moderate (0.41–0.60), good (0.61–0.80) and excellent (0.81–1) agreement. Sensitivity and specificity were used to evaluate the ability of the JEM to correctly identify exposed and unexposed individuals, respectively. The F1-score is a metric of accuracy which is less affected by imbalanced classes, a value of 1 being perfect accuracy. For each exposure, the optimal cut-off was selected based on the highest F1-score. When multiple cut-offs had equivalent F1-score, the one with the highest specificity was chosen to minimize false positives.

In addition to the group-based frequency approach, more complex methods were considered to ensure estimates were reliable: classification and regression tree (CART), random forest, and extreme gradient boosting machine. The CART method is a decision tree algorithm that has already been used to develop JEM (22–25). Random forest and extreme gradient boosting machine are ensemble learning methods that combine multiple decision trees to improve prediction accuracy (supplementary material S2) (26, 27). These methods were chosen because of their proven ability to handle complex data structures and their demonstrated performance in various contexts making them promising approaches for developing JEM. Before implementing these methods, several preprocessing steps were necessary to prepare the data. Occupational variables (1–4 digits PCS/ 1–5 digits NAF) were transformed into dummy variables, and those reporting a unique response modality were excluded from the analysis as they provided no valuable information for the models. All possible combinations of the different levels of PCS and NAF information were considered resulting in 23 different sets of occupational variables. For exposures with imbalanced data (ie, where exposed participants are substantially fewer than unexposed participants), the exposed group was overweighted using inverse probability weighting to improve the model’s ability to learn from the minority class (ie, exposed participants) and prevent bias towards the majority class (ie, unexposed participants). Multiple sets of hyperparameters were used to tune the models (supplementary material S2), and the optimal hyperparameters corresponding to those optimizing the area under the ROC curve (AUC) were automatically determined through 10-fold cross validation. For each exposure, the tuned model with the most informative set of occupational variables (defined as the set providing the higher AUC) was used to predict exposure in the test sample and evaluate performance. The exposure probabilities for each occupational group were estimated using the different models and their best set of variables and hyperparameters. The optimal cut-off for considering participants exposed was determined using the same method as for the group-based frequency method. Finally, the performances of the final models (ie, CART, random forest and extreme gradient boosting machine) with their optimal cut-off were compared with each other and with the performance of the group-based frequency approach for each exposure. Forest plots were used to provide visual representation of the performance results.

All the statistical analyses were performed using R software (v 4.3.2, R Foundation for Statistical Computing, Vienna, Austria) and CARET, rpart, ranger, xgboost, PRROC, and pROC packages.

*Alternative and simplified job exposure matrices.* Using the same methodology as described in the previous section, an alternative JEM including type of healthcare establishment (public/private) and size (<200 / ≥200 employees) in addition of NAF and PCS, and referred as “alternative JEM”, was developed and evaluated. Moreover, to enhance usability, a simplified JEM relying exclusively on job titles (PCS) was proposed using the method with the best performance results.

### Sensitivity analysis

**Figure 1:**
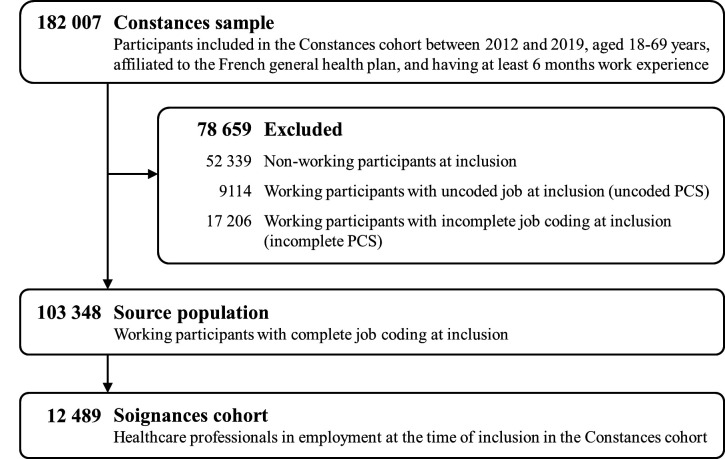
Flowchart of study population selection.

A sensitivity analysis was conducted to evaluate if including information on other occupations could provide additional context to better differentiate sub-groups within the healthcare sector and thus improve exposure estimates for healthcare professionals. The methodology detailed in the section JEM development and internal validity assessment was thus reproduced using the source cohort (N=103 348; figure 1).

## Results

### Population

In the CONSTANCES sample of 182 007 participants with ≥6 months of work experience, 103 348 had complete job coding at inclusion (figure 1). Among them, 12 489 healthcare workers in activity at the time of inclusion in the CONSTANCES cohort were identified and included in the Soignances cohort. The population was predominantly female (>80%), with a median age of 41 years [interquartile range (IQR) 32–51] (table 2). The most represented professions in the cohort were nurses (30.0%), nursing assistants (12.1%), medical technicians, assistants, and aides (11.5%), physiotherapists and rehabilitation specialists (10.9%), and physicians (9.3%).

**Table 2:** Baseline characteristics of the Soignances cohort. [IQR=interquartile range]

		Soignances cohort (N=12 489)		
		Missing data (%)	Median (IQR)	N (%)
Female		0		10 048 (80.5)
Age in years		0	41.0 (32.0; 51.0)	
**Age group**		0		
	18–29			2207 (17.7)
	30–39			3633 (29.1)
	40–49			3257 (26.1)
	50–59			2806 (22.5)
	≥ 60			586 (4.7)
	Part-time work	1.4		3087 (25.1)
	Public sector	13.2		5824 (53.7)
	Healthcare establishment with >200 employees	6.1		7930 (67.6)
**Professions**		0		
	Nurses			3744 (30.0)
	Nursing assistants			1517 (12.1)
	Medical technicians, assistants, aides			1442 (11.5)
	Physiotherapists, rehabilitation specialists			1356 (10.9)
	Physicians			1166 (9.3)
	Hospital service agents			503 (4.0)
	Clinical managers			439 (3.5)
	Psychologists, psychotherapists			378 (3.0)
	Pharmacists			343 (2.7)
	Pharmacy technicians			333 (2.7)
	Interns (medicine, dentistry, pharmacy)			305 (2.4)
	Midwives			285 (2.3)
	Dentists			241 (1.9)
	Opticians, audiologists, medical equipment specialists			226 (1.8)
	Ambulance drivers			154 (1.2)
	Veterinarians			57 (0.5)
**Occupational exposures**				
**Organizational constraints**				
	Late hours (bedtime after midnight)	0.9		1233 (10.0)
	Early hours (up before 5am)	0.9		929 (7.5)
	Sleepless nights	0.6		1264 (10.2)
	Long working hours (>10h)	0.8		3117 (25.2)
	Weekly rest <48h consecutive	1.3		1942 (15.8)
	Shift work	1.4		2641 (21.4)
	Saturday work (more than one in two)	0.7		2827 (22.8)
	Sunday work (more than one in two)	0.8		1985 (16.0)
**Biomechanical factors**				
	Time-constrained job	0.8		306 (2.5)
	Repetitive work	3.7		3162 (26.3)
	Physically difficult work	1.5		2354 (19.1)
	Physical effort at work	4.1		4822 (40.3)
	Carry heavy loads	1.3		3057 (24.8)
	Carry heavy loads (>25kg)	4.1		1647 (13.7)
	Arms above shoulder	2.3		1125 (9.2)
	Kneel or squat	1.6		4305 (35.0)
	Intense physical effort (Borg)	2.4		6186 (50.7)
**Physical factors**				
	Noise pollution	0.6		1131 (9.1)
	Noisy tools	0.8		798 (6.4)
	Ionizing radiation	4.7		1540 (12.9)
**Biological and chemical factors**				
	Formaldehyde	3.9		335 (2.8)
	Infectious risks	3.4		7241 (60.0)
	Live or dead animals	1.9		491 (4.0)
**Psychosocial factors**				
	Effort-reward imbalance ≥1.5	3.6		1866 (15.5)

Occupational exposures among healthcare workers were diverse, covering psychosocial, organizational, biomechanical, physical, and biological and chemical factors. Notably, a substantial proportion of healthcare workers experienced arduousness of work with 50.7% reporting intense physical effort, 40.3% physical effort at work, nearly one quarter declaring that they carry heavy loads (13.7% for >25kg loads) and one-fifth physically difficult work. Only 2.5% of the Soignances cohort reported exposure to time-constrained job. Noise pollution affected 9.1% of the cohort (6.4% for noisy tools) and other physical factors such as exposure to ionizing radiation affected 12.9%. Concerning organizational constraints, 25.2% of healthcare professionals experienced long working hours, 21.4% shift work, and 22.8% and 16.0% reported Saturday and Sunday work, respectively. The most frequently reported biological factor was infectious risk (60.0%), only 2.8% reported being exposed to formaldehyde. Effort-reward imbalance affected 15.5% of the cohort.

### Job exposure matrix development and internal validity

Out of the 573 occupational groups built from PCS+NAF combinations, only 117 included >10 individuals and were considered for the development of JEM Soignances (153 out of 990 for the alternative JEM). However, these groups accounted for >90% of individuals included in the Soignances cohort. Further details regarding the number of groups and individuals considered for each exposure are outlined in table 1.

**Figure 2:**
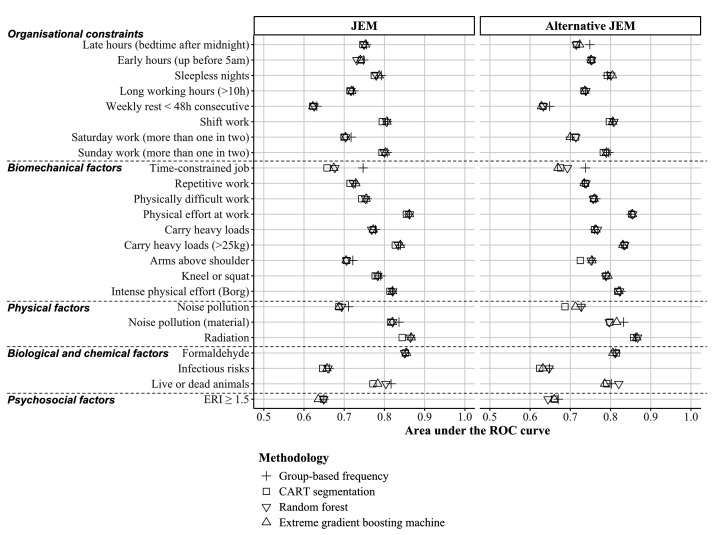
Forest plot comparing the discriminatory power (area under the ROC curve) of group-based frequency, CART, random forest, and extreme-gradient boosting machine methods for the development of JEM Soignances.

Regardless of the type of JEM (classic or alternative), for each exposure of interest the discriminatory power remained consistent across all methods used (CART, random forest, extreme-gradient boosting machine or group-based frequency; figure 2 and supplementary material table S3 for detailed performance estimates). As the results of the group-based frequency method were similar to those of the more complex models, this method was retained for the construction of the JEM. The resulting JEM (based on the full dataset) is available in the supplementary material (appendix A1 – JEM Soignances).

The performances of JEM Soignances are reported in table 3 for the determined optimal threshold (see appendix A2-A for performance estimates across different thresholds). Of the 24 exposures, 9 included in JEM Soignances offered good discriminatory power (AUC > 0.8). Among these, 3 presented moderate agreement: physical effort at work (AUC=0.861, κ=0.556), intense physical effort (Borg; AUC=0.819, κ=0.503), and exposure to ionizing radiation (AUC=0.865, κ=0.457). The remaining 6 showed fair agreement: carrying heavy loads (>25kg; AUC=0.840, κ=0.402), shift work (AUC=0.807, κ=0.383), noisy tools (AUC=0.839, κ=0.369), Sunday work (AUC=0.807, κ=0.314), and exposure to formaldehyde (AUC=0.847, κ=0.289), or working with live or dead animals (AUC=0.808, κ=0.255). The remaining 15 exposures showed either moderate discriminatory power (AUC 0.718–0.793) with fair agreement (carry heavy loads, kneel or squat, repetitive work, long working hours (>10h), physically difficult work, Saturday work), poor discriminatory power (AUC=0.661) with moderate agreement (infectious risks), or poor to moderate discriminatory power (AUC 0.631–0.793) with poor agreement (sleepless nights, arms above shoulder, late hours, noise pollution, early hours, effort-reward imbalance, weekly rest <48h consecutive, time-constrained job). With the exception of time-constrained job, repetitive work, carry heavy loads (>25 kg) and arms above shoulder which offered good specificity (0.745–0.912) and moderate sensitivity (0.425–0.641), biomechanical factors generally showed a good ability to identify exposed individuals (sensitivity=0.726–0.899) and a moderate to good ability to identify unexposed individuals (specificity=0.487–0.707). Physical, biological, and chemical factors (except infectious risks) generally exhibited good specificity values (0.860–0.981) with low to moderate ability to correctly identify exposed cases (sensitivity=0.307–0.513). Organizational constraints (except late hours and long working hours) and psychosocial risk factors generally showed good sensitivity values (0.749–0.881) but failed to minimize false positives (specificity=0.411–0.683).

**Table 3:** Performance measures of JEM Soignances (group-based frequency method). [AUC=area under the ROC curve; κ=Kappa; F1=F1-score; Spe=specificity; Sen=sensitivity]

Exposure		JEM Soignances					
		AUC	Optimal cut-off (%)	κ	F1	Spe	Sen
**Organizational constraints**							
	Late hours (bedtime after midnight)	0.752	15	0.166	0.287	0.755	0.541
	Early hours (up before 5am)	0.746	10	0.121	0.238	0.574	0.819
	Sleepless nights	0.793	15	0.203	0.330	0.683	0.749
	Long working hours (>10h)	0.721	30	0.291	0.481	0.786	0.520
	Weekly rest < 48h consecutive	0.631	15	0.103	0.327	0.411	0.820
	Shift work	0.807	30	0.383	0.564	0.674	0.846
	Saturday work (more than one in two)	0.718	25	0.238	0.492	0.531	0.814
	Sunday work (more than one in two)	0.807	25	0.314	0.487	0.633	0.881
**Biomechanical factors**							
	Time-constrained job	0.747	05	0.076	0.117	0.841	0.440
	Repetitive work	0.741	35	0.348	0.548	0.745	0.641
	Physically difficult work	0.758	25	0.272	0.474	0.653	0.726
	Physical effort at work	0.861	55	0.556	0.759	0.707	0.877
	Carry heavy loads	0.778	30	0.343	0.559	0.637	0.805
	Carry heavy loads (>25kg)	0.840	25	0.402	0.487	0.912	0.496
	Arms above shoulder	0.721	15	0.169	0.278	0.825	0.425
	Kneel or squat	0.791	30	0.328	0.641	0.487	0.899
	Intense physical effort (Borg)	0.819	50	0.503	0.792	0.623	0.873
**Physical factors**							
	Noise pollution	0.711	15	0.169	0.264	0.860	0.368
	Noisy tools	0.839	20	0.369	0.420	0.932	0.513
	Ionizing radiation	0.865	30	0.457	0.525	0.936	0.508
**Biological and chemical factors**							
	Formaldehyde	0.847	15	0.289	0.309	0.981	0.307
	Infectious risks	0.661	50	0.232	0.780	0.289	0.917
	Live or dead animals	0.808	10	0.255	0.296	0.934	0.441
**Psychosocial factors**							
	Effort-reward imbalance	0.649	0.15	0.110	0.328	0.461	0.769

The performance of the simplified JEM (job title only; appendix A2-B) was found to be unsatisfactory, characterized by poor agreement with self-reported exposures, and an inability to accurately identify exposed individuals (low specificity). The inclusion of type of healthcare establishment and size for the alternative JEM development did not notably enhance performance, as it remained largely comparable to that of JEM Soignances (appendix A2-C). While there were slight improvements for late hours (AUC=0.74 versus 0.72, κ=0.22 versus 0.17), these were considered negligible.

### Sensitivity analysis

The addition of information on extra occupations from the source cohort did not improve the accuracy of exposure estimates for healthcare professionals (supplementary material table S4).

## Discussion

This study sought to address the need for a comprehensive and accurate tool to evaluate occupational risk factors among healthcare professionals. Through the development and evaluation of a JEM derived from self-reported data, this study provides a valuable resource for both researchers and practitioners in assessing the occupational hazards encountered by healthcare workers. Most of the exposures included in the JEM reported moderate to good discriminatory power and fair-to-moderate agreement with individual self-reported data, indicative of satisfactory performance. The performance of the JEM was particularly good for biomechanical, physical, biological, and chemical factors, and especially for indicators of arduousness of work (intense physical effort, physical effort at work, carry heavy loads, physically difficult work) and exposure to ionizing radiation, while infectious risk surprisingly reported moderate performance. Psychosocial and organizational constraints provided lower performance estimates. These findings were expected concerning psychosocial factors, as perceived effort-reward imbalance, a proxy of work stress, can be strongly influenced by individual factors (eg, resilience, private life), contextual factors (eg, leadership issues), and specificities of workplace organization factors not captured by the JEM (28–30). Nevertheless, the substantial proportion of healthcare professionals reporting high effort-reward imbalance (16%) is consistent with the demanding nature of their occupational environment. Conversely, these results were more surprising regarding organizational factors, some were rarer than expected (eg, early or late hours), other were common but spread across various occupational groups (eg, shift work) highlighting the absence of some important discriminatory variables (eg, hospital department). Indeed, some exposures garnered particular attention within specific care contexts, such as stressful situations like violence in emergency departments (31), exposure to noise in operating rooms (32), or ionizing radiations in interventional radiology departments (33). Some of these risks are mitigated by preventive measures (eg, radiation protection) that, like variation across hospital departments, cannot be considered in our study.

JEM Soignances is, to our knowledge, the first JEM to focus exclusively on all healthcare workers within a broad range of exposures. Previous efforts to develop a JEM based on expert knowledge within hospital settings have been attempted but have not resulted in the dissemination of a JEM (34). Other JEM were specifically designed for specific risks or exposures. First, a US JEM focused on occupational risk factors for asthma in healthcare workers (35). None of the exposures considered in the US JEM (cleaning products, powdered latex gloves, aerosolized medications, adhesives/solvents/gases, and sensitizing metals) covered our exposures of interest. Second, a French nurse-specific JEM focused on exposure to disinfectants (36). The authors of the latter pointed out the limitations of JEM regarding the heterogeneity of tasks within the same job groups and also proposed a job-task exposure matrix (JTEM). The exposure estimates for formaldehyde obtained in the Soignances JEM never exceeded 5% except for specialized nurses in hospital (22.1%) and were globally similar to those of the nurse-specific JEM and JTEM for nurses working in emergency room, outpatient or community, and education or administration. However, in the nurse-specific JEM, the reported percentage for nurses working in operating room was 30%, highlighting the complementarity of our general approach, which provides an overall view of the exposure of healthcare professionals, and of their specific approach reporting more accurate estimates within specific care contexts.

Compared to general population JEM, JEM Soignances offers good performance measures. The French gender-specific JEM Constances, focusing on physical risk factors, was developed using a large sample from the CONSTANCES cohort (21). The authors reported slightly lower overall discriminatory power and agreement with self-reported exposure compared to JEM Soignances for intense physical effort (AUC=0.75–0.76 versus 0.82, κ=0.33–0.35 versus 0.50), carrying heavy loads (>25kg) (AUC=0.62–0.73 versus 0.84, κ=0.23–0.29 versus 0.40), and repetitive work (AUC=0.67–0.68 versus 0.74, κ=0.26 versus 0.35). However, JEM Constances performed best for arms up (AUC=0.67–0.71 versus 0.72, κ=0.26–0.28 versus 0.17) and kneeling/squatting postures (AUC=0.80–0.81 versus 0.79, κ=0.46–0.48 versus 0.33). Similar results were found with a Finnish JEM focused on physical risk factors in low back pain (19) and a Norwegian JEM focused on mechanical and psychosocial exposures (20). The first one reported similar performance for intense physical effort (κ=0.41–0.52), while the second reported poorer results (AUC=0.65–0.74, κ=0.23–0.34). Both reported poorer performance for carrying heavy loads (κ=0.26–0.28 and AUC=0.72–0.73, κ=0.27–0.35 respectively) and better performance for postures such as kneeling/squatting (κ=0.35–0.53 and AUC=0.69–0.77, κ=0.34–0.51 respectively) or arms up postures (κ=0.16–0.47 and AUC=0.67–0.77, κ= 0.25–0.42 respectively). Similarly, a Danish JEM (37), which also examined biomechanical and organizational factors, reported comparable results, with better performance for “physical work demands” (AUC=0.85–0.87) than for “quantitative demands” (AUC=0.62–0.64), echoing the trend observed in our study where biomechanical factors outperformed organizational ones. Regarding psychosocial exposures, the Norwegian JEM and a French JEM for psychosocial factors based on the SUMER survey reported more precise exposures with better performance estimates (20, 38). However, the application of these JEM might present challenges in healthcare professionals’ populations. Firstly, relying solely on job titles seems inadequate for healthcare professionals due to the varied nature of their tasks across different sectors of activity. Secondly, as indicated by the sensitivity analysis, incorporating data from other occupations does not enhance the accuracy of exposure estimates for healthcare workers.

### Strengths and limitations

This study is the first to provide a JEM specific to healthcare workers. Moreover, a large cohort of healthcare professionals was used, allowing JEM Soignances to cover a wide range of exposures and numerous occupational groups. As healthcare professionals’ tasks can vary greatly depending on the sector in which they work, this JEM not only considers the specific job of healthcare professionals but also incorporates the sector of activity in which they are employed. JEM Soignances will facilitate future studies aimed at assessing healthcare professionals’ health to take occupational exposures into account.

This study has weaknesses inherent to the nature of the data used. CONSTANCES relies on voluntary participation, which can introduce selection bias and affect the representativeness of the sample. Participants in voluntary cohorts, such as CONSTANCES, tend to be healthier and have a higher socio-economic status than the general population, as found in other general cohorts (39, 40). In the current study, we focused on healthcare workers. However, due to the lack of data on healthcare workers in France, we were unable to directly account for selection biases. As a result, it is challenging to ensure the representativeness of our sample compared to the broader population of healthcare professionals in France. Notably, healthcare workers are overrepresented among active participants in the CONSTANCES cohort (approximately 10% compared to 2–3% in the French active population). Although these limitations are notable, another JEM based on these data showed good generalizability on US data (41). Moreover, the use of self-reported data may introduce classification bias potentially leading to the over- or underestimation of exposure levels (42). Additionally, the absence of information on hospital department or the presence of preventive measures in the CONSTANCES dataset limits the ability of this JEM to account for such factors, which could influence exposure estimates. This study also faced classical limitations of JEM. Firstly, there are no gold standard to formally validate the JEM. Secondly, difficulties in coding occupations may result in incomplete data, leading to the exclusion of participants. Additionally, occupational groups with ≤10 participants have been excluded. While this exclusion is known to help maintain precision in the estimates, there is no consensus on the minimum number of participants to retain (43). In our study, <10% of the population was excluded. Thirdly, JEM measurements represent averaged measurements and may not accurately reflect individual-level exposures. Heterogeneity of exposures within a given occupational group may lead to non-differential misclassification of estimated individual exposures (8). However, JEM have shown good performance at population level (44). Finally, exposures may vary over time, especially when comparing pre- and post-COVID-19 periods, and across countries due to major differences healthcare systems structures. Nonetheless, the universal constraints faced by healthcare professionals worldwide underscore the importance of this JEM and the need for similar initiatives in other countries.

### Concluding remarks

In conclusion, a specific healthcare workers’ JEM – JEM Soignances – was constructed and its internal validity evaluated. This JEM covers various facets of healthcare professionals’ occupational exposome, including exposure to biomechanical, physical, biological, chemical, psychosocial, and organizational factors. This JEM offers applications for future research focused on healthcare workers and is particularly suited for use with large database where only job titles and activity sectors are available.

Subsequent studies should evaluate the external validity of JEM Soignances by comparing exposure estimates with those from existing JEM for various occupational groups. Its predictive validity will be assessed by investigating established associations between occupational exposures and health outcomes such as the association between long working hours and strokes. Additionally, to ensure the JEM remains accurate in evolving healthcare environments, including post-pandemic conditions, further validation with external data is crucial. While this JEM is valuable for understanding the multifaceted nature of occupational hazards in healthcare settings, additional research is necessary to consider the concurrent exposure to these multiple hazards.

## Supplementary material

Appendices A1-A2

Supplementary material
