# Supplementary material for: Development and validation of a French job-exposure matrix for healthcare workers: JEM Soignances
Source: Scand J Work Environ Health. 2024 Nov 28;50(8):653–64. doi: 10.5271/sjweh.4194 (PMC11626462; doi:10.5271/sjweh.4194)
Supplement: Supplementary material [file SJWEH-50-653-S002.pdf]

Development and validation of a French job-exposure matrix for healthcare workers: JEM Soignances[1]

by Allison Singier, PhD,[2] Marc Fadel, MD, Fabien Gilbert, MSc, Soignances group, Ester-MESuRS collaboration on occupational risks, Laura Temime, PhD, Marie Zins, MD, PhD, Alexis Descatha, MD, PhD

1. Supplementary material
2. Correspondence to: Allison Singier, INSERM U1085 - Irset, ESTER team, Faculté de santé - Département Médecine, 28 rue Roger Amsler, CS 74521, F-49045 Angers cedex 1, France. [E-mail address: allison.singier@inserm.fr]

Other Appendices available at https://www.sjweh.fi/article/4194

- A1. JEM Soignances
- A2. Detailed performance estimates for JEM Soignances and the two other developed JEMs (simplified and alternative) at different thresholds

S1. Proposed crosswalk between PCS-2003 and ISCO-2008

PCS label in French	PCS label in English	PCS-2003	ISCO-2008	ISCO label in English	ISCO label in French
Médecins libéraux spécialistes	Self-employed specialist medical practitioners	**311A**	**2212**	Specialist medical practitioners	Médecins spécialistes
Médecins libéraux généralistes	Self-employed generalist medical practitioners	**311B**	**2211**	Generalist medical practitioners	Médecins généralistes
Chirurgiens dentistes (libéraux ou salariés)	Dental Surgeons (self-employed or employees)	**311C**	**2261**	Dentists	Dentistes
Psychologues, psychanalystes, psychothérapeutes (non médecins)	Psychologists, psychoanalyst, psychotherapist (non medical)	**311D**	**2634**	Psychologists	Psychologues
Vétérinaires (libéraux ou salariés)	Veterinarians (self-employed or employees)	**311E**	**2250**	Veterinarians	Vétérinaires
Médecins hospitaliers sans activité libérale	Specialist medical practitioners in hospitals	**344A**	**2212**	Specialist medical practitioners	Médecins spécialistes
Médecins salariés non hospitaliers	Generalist medical practitioners (employees)	**344B**	**2211**	Generalist medical practitioners	Médecins généralistes
Internes en médecine, odontologie et pharmacie	Resident in medicine, dentistry and pharmacy	**344C**	**2211**	Generalist medical practitioners	Praticiens paramédicaux
Pharmaciens salariés	Employed pharmacists	**344D**	**2262**	Pharmacists	Pharmaciens
Cadres infirmiers et assimilés	Nursing managers and equivalent	**431A**	**1342**	Health services managers	Directeurs et cadres dirigeants, services de santé
Infirmiers psychiatriques	Psychiatric nurses	**431B**	**2221**	Nursing professionals	Infirmiers qualifiés
Puéricultrices	Pediatric nurses	**431C**	**2221**	Nursing professionals	Infirmiers qualifiés
Infirmiers spécialisés (autres qu'infirmiers psychiatriques et puéricultrices)	Specialized nurses (other than psychiatric and pediatric nurses	**431D**	**2221**	Nursing professionals	Infirmiers qualifiés
Sages-femmes (libérales ou salariées)	Midwives (self-employed or employees)	**431E**	**2222**	Midwifery professionals	Sages-femmes
Infirmiers en soins généraux, salariés	Nurses (employees)	**431F**	**3221**	Nursing associate professionals	Personnel infirmier
Infirmiers libéraux	Nurses (self-employed)	**431G**	**3221**	Nursing associate professionals	Personnel infirmier
Masseurs-kinésithérapeutes rééducateurs, libéraux	Physiotherapists, self-employed	**432A**	**2264**	Physiotherapists	Kinésithérapeutes et physiothérapeutes
Masseurs-kinésithérapeutes rééducateurs, salariés	Physiotherapists, employees	**432B**	**2264**	Physiotherapists	Kinésithérapeutes et physiothérapeutes
Autres spécialistes de la rééducation, libéraux	Other rehabilitation specialists, self-employed	**432C**	**2269**	Health professionals not elsewhere classified	Spécialistes de la santé non classés ailleurs

Autres spécialistes de la rééducation, salariés	Other rehabilitation specialists, employees	**432D**	**2269**	Health professionals not elsewhere classified	Spécialistes de la santé non classés ailleurs
Techniciens médicaux	Medical technicians	**433A**	**3211**	Medical imaging and therapeutic equipment technicians	Techniciens d'appareils électromédicaux
Opticiens lunetiers et audioprothésistes (indépendants et salariés)	Opticians and hearing aid specialist (self-employed and employed)	**433B**	**2267**	Optometrists and ophthalmic opticians	Opticiens et optométristes
Autres spécialistes de l'appareillage médical (indépendants et salariés)	Other specialist in medical equipment (self-employed and employed)	**433C**	**3214**	Medical and dental prosthetic technicians	Techniciens de prothèses médicales et dentaires
Préparateurs en pharmacie	Pharmacy assistant	**433D**	**3213**	Pharmaceutical technicians and assistants	Techniciens et assistants pharmaciens et préparateurs en pharmacie
Agents de service hospitaliers (de la fonction publique ou du secteur privé)	Hospital service agents (public service or private sector)	**525D**	**9112**	Cleaners and helpers in offices, hotels and other establishments	Agents d'entretien dans les bureaux, les hôtels et autres établissements

There is no official equivalence between the French socio-professional categories (PCS) and the International Standard Classification of Occupations (ISCO). The provided crosswalk represents an indicative attempt to match the closest possible categories between the two classification systems. However, these correspondences may not fully capture the specific nuances of each classification, due to particular aspects of the French workplace environment, workforce and economic structure. We therefore advise using these correspondences with caution and not treating them as strict or universally valid equivalents.

S2. Additional details regarding the development and validation of JEM Soignances

All models were trained, and their performance evaluated using R software (v 4.3.2, R Foundation for Statistical Computing, Vienna, Austria) and the CARET package.

Three types of models were used for the purpose of this study: Classification And Regression Tree (CART), random forest, and extreme gradient boosting machine. Briefly:

- CART models are simple decision tree models that divide data into subsets based on different characteristics. These models can be represented as (more or less complex) tree structures where each node represents a decision based on a feature, and the leaves represent the results. While easy to interpret, CART models are prone to overfitting and may not generalize well to new data.

- Random forests are ensembles of multiple decision trees, where each tree is trained on a random subset of the data. The final prediction is based on the most frequent result (for classification) across the set of trees. Compared to CART models, random forests are less prone to overfitting and improve accuracy by aggregating the results of multiple trees.

- Extreme Gradient Boosting Machine are other ensemble models, but unlike random forests, it builds trees sequentially. Each new tree is designed to correct the errors made by the previous ones. This "boosting" increases accuracy, particularly for complex datasets, but can make the model harder to interpret compared to the models mentioned above.

Details about the hyperparameters used to tune CART, random forest and extreme gradient boosting machine models are outlined below.

For CART modelling, the rpart package was used. The model's tuning involved a single hyperparameter, the complexity parameter *cp*. A list of 10 values ranging from 0 to 0.01 in increments of 0.001 was used.

For random forest modelling, the ranger package was used. The number of trees was set to 250, and three hyperparameters were used to tune the model:

- *mtry* the number of random predictors selected (i.e. number of variables to consider for splitting each node),
- *splitrule* the splitting rule,

- *min.node.size* the minimum size of terminal nodes.

A grid combining all possible combinations of 5 *mtry* values randomly generated using CARET's var_seq function, 2 types of splitting (gini impurity index or extremely randomised trees), and 2 values for *min.node.size* (10 or 20).

For extreme gradient boosting machine modelling, the xgbTree method from the CARET package (based on xgBoost package) was used. Seven hyperparameters were considered for tuning:

- *nrounds* the number of boosting iterations (or trees),
- *max_depth* the maximum tree depth,
- *eta* the learning rate,
- *gamma* the minimum loss reduction required to make a further partition on a leaf node of the tree,
- *colsample_bytree* the proportion of columns used to build each tree,
- *min_child_weight* the minimum sum of instance weight required in a child node,
- *subsample* the proportion of instances used to build each tree.

Given the large number of hyperparameters, CARET's tuneLength function was used to control the number of randomly generated combinations for this tuning parameter search.

S3. Detailed performance estimates (Soignances cohort)

Table S3. Detailed performance estimates for group-based frequency, CART, random forest, and extreme-gradient boosting machine methods for JEM construction using Soignances data

Exposure	Group-based frequency		CART segmentation		Random forest		Extreme-gradient boosting machine	
	AUC	AUPRC	AUC	AUPRC	AUC	AUPRC	AUC	AUPRC
Organisational constraints								
Late hours (bedtime after midnight)	0.75	0.22	0.75	0.21	0.75	0.21	0.76	0.22
Early hours (up before 5am)	0.75	0.16	0.75	0.16	0.74	0.15	0.75	0.16
Sleepless nights	0.79	0.30	0.78	0.27	0.78	0.30	0.79	0.30
Long working hours (>10h)	0.72	0.44	0.72	0.42	0.72	0.41	0.72	0.43
Weekly rest < 48h consecutive	0.63	0.21	0.63	0.21	0.63	0.21	0.63	0.21
Shift work	0.81	0.45	0.80	0.44	0.81	0.44	0.81	0.44
Saturday work (more than one in two)	0.72	0.39	0.70	0.38	0.71	0.39	0.71	0.38
Sunday work (more than one in two)	0.81	0.39	0.80	0.36	0.81	0.37	0.81	0.38
Biomechanical factors								
Time-constrained job	0.75	0.07	0.66	0.05	0.68	0.05	0.68	0.06
Repetitive work	0.74	0.50	0.72	0.46	0.73	0.47	0.73	0.48
Physically difficult work	0.76	0.39	0.75	0.38	0.76	0.40	0.76	0.40
Physical effort at work	0.86	0.79	0.86	0.77	0.87	0.79	0.87	0.79
Carry heavy loads	0.78	0.49	0.78	0.48	0.77	0.46	0.78	0.48
Carry heavy loads (>25kg)	0.84	0.43	0.83	0.37	0.84	0.42	0.84	0.43
Arms above shoulder	0.72	0.21	0.71	0.19	0.71	0.20	0.71	0.20
Kneel or squat	0.79	0.68	0.78	0.66	0.79	0.67	0.79	0.66
Intense physical effort (Borg)	0.82	0.82	0.82	0.81	0.83	0.82	0.83	0.82
Physical factors								
Noise pollution	0.71	0.20	0.69	0.19	0.70	0.20	0.69	0.19
Noisy tools	0.84	0.33	0.82	0.29	0.83	0.31	0.82	0.31

Ionising radiation	0.87	0.52	0.85	0.44	0.87	0.52	0.87	0.54
Biological and chemical factors								
Formaldehyde	0.85	0.21	0.86	0.21	0.85	0.21	0.86	0.22
Infectious risks	0.66	0.73	0.65	0.73	0.66	0.73	0.66	0.73
Live or dead animals	0.81	0.27	0.78	0.24	0.81	0.26	0.79	0.26
Psychosocial factors								
Effort-reward imbalance	0.65	0.23	0.65	0.24	0.65	0.23	0.64	0.23

AUC: area under the ROC curve, AUPRC: area under the Precision-Recall curve

Compared to AUC, AUPRC provides a complementary measure of model performance in class-imbalanced cases, focusing on precision (the proportion of true positives among predicted positives) and recall (the proportion of actual positives correctly identified) rather than sensitivity and specificity.

S4. Detailed performance estimates (source cohort)

Table S4. Detailed performance estimates of CART, random forest, and extreme-gradient boosting machine methods for JEM construction using source cohort data

Exposure	CART segmentation		Random forest		Extreme-gradient boosting machine	
	AUC	AUPRC	AUC	AUPRC	AUC	AUPRC
Organisational constraints						
Late hours (bedtime after midnight)	0.68	0.16	0.76	0.21	0.76	0.22
Early hours (up before 5am)	0.51	0.08	0.75	0.17	0.75	0.17
Sleepless nights	0.72	0.17	0.80	0.30	0.79	0.30
Long working hours (>10h)	0.73	0.42	0.73	0.45	0.73	0.45
Weekly rest < 48h consecutive	0.57	0.18	0.63	0.21	0.62	0.21
Shift work	0.80	0.42	0.82	0.46	0.81	0.45
Saturday work (more than one in two)	0.72	0.40	0.72	0.40	0.71	0.40
Sunday work (more than one in two)	0.80	0.37	0.81	0.39	0.80	0.37
Biomechanical factors						
Time-constrained job	0.68	0.06	0.69	0.06	0.71	0.06
Repetitive work	0.73	0.47	0.74	0.48	0.74	0.48
Physically difficult work	0.76	0.38	0.76	0.40	0.76	0.40
Physical effort at work	0.85	0.76	0.87	0.79	0.87	0.79
Carry heavy loads	0.79	0.49	0.78	0.49	0.78	0.48
Carry heavy loads (>25kg)	0.85	0.41	0.85	0.43	0.85	0.42
Arms above shoulder	0.73	0.20	0.69	0.19	0.73	0.21
Kneel or squat	0.79	0.68	0.80	0.68	0.79	0.67
Intense physical effort (Borg)	0.81	0.78	0.83	0.82	0.83	0.82
Physical factors						
Noise pollution	0.71	0.21	0.71	0.21	0.71	0.21
Noisy tools	0.84	0.31	0.85	0.33	0.84	0.33
Ionising radiation	0.86	0.49	0.87	0.54	0.87	0.54
Biological and chemical factors						
Formaldehyde	0.84	0.20	0.85	0.20	0.86	0.22
Infectious risks	0.63	0.69	0.67	0.74	0.66	0.74
Live or dead animals	0.80	0.26	0.80	0.26	0.80	0.27
Psychosocial factors						
Effort-reward imbalance	0.63	0.22	0.63	0.21	0.65	0.23

AUC: area under the ROC curve, AUPRC: area under the Precision-Recall curve

Compared to AUC, AUPRC provides a complementary measure of model performance in class-imbalanced cases, focusing on precision (the proportion of true positives among predicted positives) and recall (the proportion of actual positives correctly identified) rather than sensitivity and specificity.
